# Variation in Tap Water Mineral Content in the United Kingdom: Is It Relevant for Kidney Stone Disease?

**DOI:** 10.3390/jcm11175118

**Published:** 2022-08-30

**Authors:** Kirolos G. F. T. Michael, Bhaskar K. Somani

**Affiliations:** 1 Northern Care Alliance NHS Foundation Trust, Salford M6 8HD, UK; 2 Department of Urology, University Hospital Southampton, Southampton SO16 6YD, UK

**Keywords:** urolithiasis, kidney calculi, tap water, mineral composition, kidney stones

## Abstract

Introduction: The dissolved mineral content of drinking water can modify a number of excreted urinary parameters, with potential implications for kidney stone disease (KSD). The aim of this study is to investigate the variation in the mineral content of tap drinking water in the United Kingdom and discuss its implications for KSD. Methods: The mineral composition of tap water from cities across the United Kingdom was ascertained from publicly available water quality reports issued by local water supply companies using civic centre postcodes during 2021. Water variables, reported as 12-monthly average values, included total water hardness and concentrations of calcium, magnesium, sodium and sulphate. An unpaired t-test was undertaken to assess for regional differences in water composition across the United Kingdom. Results: Water composition data were available for 66 out of 76 cities in the United Kingdom: 45 in England, 8 in Scotland, 7 in Wales and 6 in Northern Ireland. The median water hardness in the United Kingdom was 120.59 mg/L CaCO_3_ equivalent (range 16.02–331.50), while the median concentrations of calcium, magnesium, sodium and sulphate were 30.46 mg/L (range 5.35–128.0), 3.62 mg/L (range 0.59–31.80), 14.72 mg/L (range 2.98–57.80) and 25.36 mg/L (range 2.86–112.43), respectively. Tap water in England was markedly harder than in Scotland (192.90 mg/L vs. 32.87 mg/L as CaCO_3_ equivalent; *p* < 0.001), which overall had the softest tap water with the lowest mineral content in the United Kingdom. Within England, the North West had the softest tap water, while the South East had the hardest water (70.00 mg/L vs. 285.75 mg/L as CaCO_3_ equivalent). Conclusions: Tap water mineral content varies significantly across the United Kingdom. Depending on where one lives, drinking 2–3 L of tap water can contribute over one-third of recommended daily calcium and magnesium requirements, with possible implications for KSD incidence and recurrence.

## 1. Introduction

The aetiology of kidney stone disease (KSD) is complex and is the product of the intricate interplay between dietary, lifestyle, environmental and genetic factors which predispose individuals to disease [1]. In the United Kingdom, the prevalence of KSD is rising, with an estimated 1 in 7 individuals requiring intervention during their lifetime, posing a substantial burden to health services [2,3]. There is therefore great impetus for investigating factors implicated in KSD, which may lead to more specific preventative strategies.

At present, the mainstay of KSD prevention is to advise patients to increase their daily fluid intake [4,5]. Nevertheless, whether or not the type of fluid matters is still debatable. Amongst studies conducted to investigate whether any type of water is superior for patients with KSD, there is a weak consensus that mineral-rich water may result in favourable changes to urine composition, which may reduce the risk of calcium stone formation [6]. For this reason, a number of studies have sought to compare the mineral composition of drinking water, whether bottled or supplied through taps, to further study the association between water composition and KSD [7,8,9]. 

Drinking water supplied through taps is derived from different sources depending on the region, leading to variations in its dissolved mineral content. [10] The “hardness” of tap water reflects the quantity of dissolved metal ions, principally calcium and magnesium [11]. Given the recognised implications of drinking water on human health, most countries monitor and tightly regulate tap water quality and composition, though recommended ranges and maximum values are largely not based on research [12]. In the United Kingdom, governmental studies have revealed that up to 97% of adults drink tap water, with the average adult consuming 1.3 L of tap water per day, accounting for nearly two-thirds of daily fluid consumption in England and Wales [13]. Given these findings, the aim of this study is to investigate the variation in tap water composition across the United Kingdom and describe potential implications for KSD.

## 2. Materials and Methods

The mineral composition of tap water during 2021 across all officially designated cities in the United Kingdom was investigated from online, publicly available water quality reports obtained from the local water supply company using the postcode of the city hall or civic centre, as a representative of the area. Where reports were not available online, water supply companies were contacted directly to request these. Cities that did not have water quality reports covering 2021 were excluded. Water variables collected included total water hardness, in addition to the concentrations of calcium, magnesium, sodium and sulphate where available. Values obtained represent an average value over a 12-month period for a given area. Potassium and bicarbonate concentrations were not included due to insufficient data across the regions to enable comparison. 

To determine whether tap water mineral composition varies significantly between regions of the United Kingdom, a pairwise comparison of mean water variables was undertaken between constituent countries in the United Kingdom. Statistical analysis was undertaken using SPSS Statistics for Windows, version 25 (IBM Corp., Armonk, NY, USA), and statistical significance was determined at the ≤0.05 level.

## 3. Results

### 3.1. Comparison of Water Composition across Constituent Countries in the United Kingdom

In total, 66 out of 76 cities in the United Kingdom were included in this study: 45 in England, 8 in Scotland, 7 in Wales and 6 in Northern Ireland. Tap water was supplied to these by 17 different water supply companies across the United Kingdom. 

The median water hardness in the United Kingdom was 120.59 mg/L CaCO_3_ equivalent (range: 16.02–331.50). The median concentrations of calcium, magnesium, sodium and sulphate were 30.46 mg/L (range: 5.35–128.0), 3.62 mg/L (range: 0.59–31.80), 14.72 mg/L (range: 2.98–57.80) and 25.36 mg/L (range: 2.86–112.43), respectively. 

**Figure 1:**
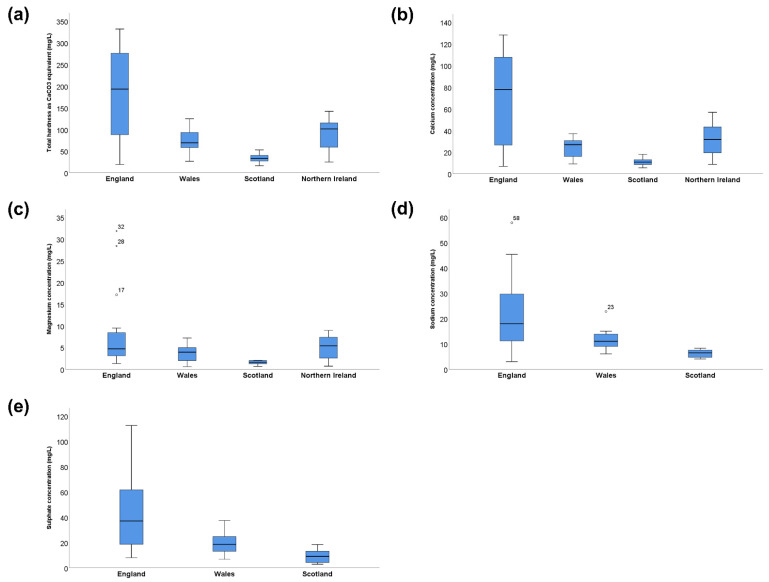
Distribution of the mineral composition of tap water across the United Kingdom. Mineral composition of tap water by country (mg/L). (**a**) Total water hardness as CaCO3 equivalent (**b**) Calcium concentration (**c**) Magnesium concentration (**d**) Sodium concentration (**e**) Sulphate concentration. ○ Outlier (value > 1.5 IQR); ★ Extreme outlier (value > 3 IQR). N.D. denotes no data available.

A comparison of the median values and ranges of water composition variables of interest between countries in the United Kingdom is presented in Table 1 and Figure 1. Compared to Scotland, which had the lowest mineral content, tap water in England was significantly harder (192.90 mg/L vs. 32.87 mg/L as CaCO_3_ equivalent) and had a higher concentration of calcium (77.56 mg/L vs. 10.69 mg/L), magnesium (4.65 mg/L vs. 1.59 mg/L), sodium (17.90 mg/L vs. 6.39 mg/L) and sulphate (37.00 mg/L vs. 9.07 mg/L) when comparing median values. A pairwise comparison of mean water variables revealed statistically significant differences between water composition values across the United Kingdom (Table 2).

### 3.2. Regional Variation in Tap Water Composition across England

**Figure 2:**
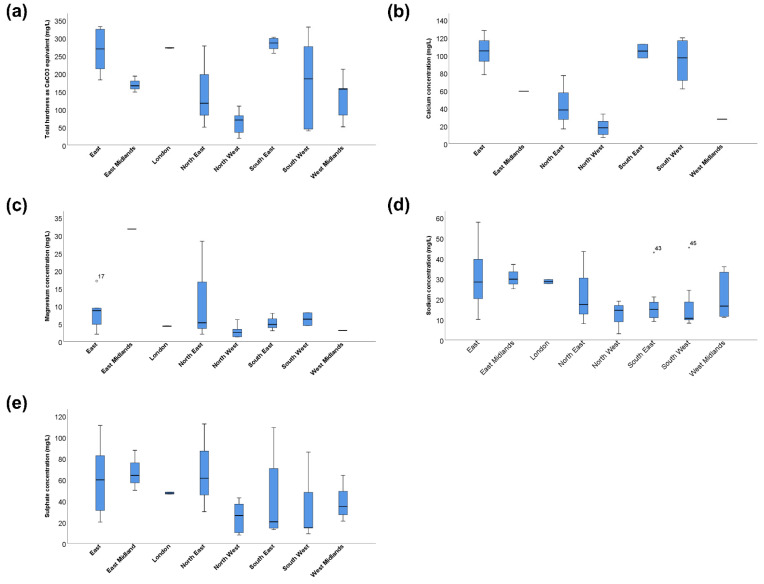
Distribution of the mineral composition of tap water across England Mineral composition of tap water by region (mg/L). (**a**) Total water hardness as CaCO3 equivalent (**b**) Calcium concentration (**c**) Magnesium concentration (**d**) Sodium concentration (**e**) Sulphate concentration. ○ Outlier (value > 1.5 IQR); ★ Extreme outlier (value > 3 IQR).

Given the wide range of water variables in England, a comparison of tap water composition across the different regions of England was undertaken. Cities in eight out of the nine regions in England had freely available water quality reports from 2021, with no cities in the Yorkshire and the Humber region reporting water composition beyond 2020 at the time of the investigation. The differences in water composition across the eight regions are presented in Table 3 and Figure 2. Even within England, there was a four-fold difference between the region with the hardest tap water (South East) and the region with the softest water (North West). Similarly, there was approximately a six-fold difference between the region with the highest calcium concentration (East) and the North West, as well as a near the 13-fold difference between the region with the highest magnesium concentration (East Midlands) and the North West.

### 3.3. Comparison of Bottled Water and Tap Water

Tap water mineral content in the United Kingdom was compared to that of commonly available bottled water brands comprising 11 brands of still water and 6 of sparkling water, as previously described by Stoots et al. (Table 4) [8]. Compared to bottled still and sparkling water from popular brands in the United Kingdom, tap water had a lower median calcium and magnesium concentration but a greater range in these values overall. By contrast, tap water had a higher sodium and sulphate content compared to bottled water. 

## 4. Discussion

### 4.1. Findings from Our Study

Our study described the variation in the mineral composition of drinking water supplied through taps across the United Kingdom. We found significant regional variation in tap water hardness and calcium, magnesium, sodium and sulphate concentrations of tap water. Notably, we report a 24-fold and 54-fold difference between the maximum and minimum tap water calcium and magnesium concentrations across regions of the United Kingdom. Interestingly, whilst bottled water, on average, had higher concentrations of most minerals of interest, the ranges of these values for tap water were larger. As far as the authors are aware, this study is the first to compare tap water mineral content across the different cities and regions of the United Kingdom.

### 4.2. Mineral Content and Pathogenesis of KSD

A number of minerals present in drinking water likely play a role in the pathogenesis of KSD, particularly calcium, magnesium and sodium. At present, the literature is in agreement that moderate calcium intake is protective against KSD, though supplemental calcium may not be beneficial and could, on the contrary, increase the risk of calcium nephrolithiasis, especially if taken separately from meals [14]. Likewise, the role of magnesium in protecting against KSD is widely recognised, while sulphate may be protective against calcium nephrolithiasis by reducing ionised urinary calcium and supersaturation of calcium salts [6,15,16,17]. Conversely, sodium in the form of salt (sodium chloride) is a well-established risk factor for calcium nephrolithiasis, and it is a routine clinical practice to counsel patients at risk of KSD to reduce their salt intake [18]. 

### 4.3. Comparison with Previous Studies

Several studies investigating tap water mineral variation have been undertaken, with comparable findings. In the Flanders region of Belgium, tap water mineral content was found to vary significantly, with a 10-fold and 12-fold difference between the highest and lowest calcium and magnesium concentrations with similar maximum values reported compared to the United Kingdom [9]. Similarly, in Australia, tap water calcium was found to vary regionally by a factor of 15.6, while magnesium varied by a factor of 10.7, though unlike in Flanders, the mineral content of tap water overall was significantly lower compared to the United Kingdom, with the maximum calcium and magnesium concentrations being approximately 6-times and 3-times lower [19]. In North America, one study found a 42-fold difference in tap water calcium concentration, while there was a 48-fold difference in magnesium concentration between regions with the highest and lowest concentrations [20]. It should be noted that these comparisons are, in most cases, between regions with the highest and lowest 12-monthly average figures; thus, differences are likely to be even larger if day-to-day variations are considered. 

### 4.4. Implications for Clinical Practice

For adults living in the United Kingdom, the recommended daily intake for calcium is 700 mg/d, while for magnesium, it is 300 mg/d (males) or 270 mg (females); for sodium, it is 2400 mg/d [21]. Our study found that depending on where one lives, drinking 2 L of tap water can contribute 1.5–36.6% of recommended daily calcium intake and 0.4–23.6% of daily magnesium intake, making tap water a significant but often overlooked source of these minerals. By contrast, tap water contributes 0.2–4.7% of daily sodium intake, which is relatively insignificant compared to other dietary sources. Furthermore, the proportion of calcium and magnesium derived from tap water is likely to be even higher for KSD patients, who will often be advised to drink up to 3 L of fluid per day. The British Association of Urological Surgeons (BAUS) includes advice on calcium intake in its “dietary advice for stone formers” patient information leaflet, highlighting that daily intake of up to 1,000 mg of calcium is safe whilst also detailing the calcium content of a number of dairy products for reference [22]. Our finding that tap water in the United Kingdom can be a significant contributor to daily calcium intake raises an interesting question: should clinicians routinely advise KSD patients to be mindful of the mineral content of their tap water? Similarly, should such advice be included on patient information leaflets? 

Having recognised that significant variations in the mineral content of tap water exist regionally and globally and that tap water can be a significant contributor to daily calcium and magnesium intake, the question then becomes whether these regional variations are of clinical significance when it comes to KSD incidence and recurrence. A number of interventional studies have demonstrated that consumption of drinking water with different mineral compositions can result in changes to excreted urinary calcium, magnesium and citrate levels as well as urinary pH, with a weak consensus in the literature favouring hard, mineral-rich water for patients at risk of KSD [6]. When compared to tap water in our study, the mineral content of different types of water included in these study protocols was, for the most part, within the ranges of total hardness, calcium and magnesium levels in tap water in the United Kingdom, although the maximum calcium concentrations in some of the studies were significantly higher, being derived from bottled mineral water [23,24,25]. It can therefore be hypothesised that variation in the mineral content of tap water in the United Kingdom may translate into variations in excreted urinary parameters of key promoting and inhibitory lithogenic factors. This is supported by a large North American study which found that 24-h urine calcium, magnesium and citrate increased with tap water hardness [26]. Nevertheless, the same study did not find large differences in the number of lifetime KSD episodes between those living in regions with soft versus hard water, though dietary, metabolic and other environmental risk factors for urolithiasis were not controlled for. Moreover, in Iran, a weak inverse correlation was demonstrated between tap water magnesium concentration and KSD incidence, further raising the possibility that tap water variations may be implicated in KSD incidence [27].

### 4.5. Limitations and Future Direction

A number of limitations are present in our study. Since water composition data were derived from 19 different water supply companies providing for the 66 cities included in our study, there was a degree of heterogeneity in how tap water quality and composition were reported between companies. Though all values were reported as a 12-monthly average, with most companies reporting mean values, for others, it was not clear what kind of average was reported. Furthermore, a number of water supply companies did not report all variables of interest in this study, though every company reported total water hardness, and the vast majority reported calcium and magnesium levels. Few reports included pH, bicarbonate and potassium levels and hence were not included in our study since meaningful comparisons between regions could not be undertaken. While our study described variations in tap water mineral composition, it did not relate this to KSD incidence or recurrence. Finally, we considered the mineral content of tap water in light of KSD; however, there are a number of other conditions, including mineral bone disease, that may be impacted by drinking water mineral composition, which should not be neglected when advising patients on the optimal type of water [28]. To further investigate the association between tap water and KSD, future studies should explore whether variation in tap water mineral content correlates with KSD incidence. Additionally, it would be interesting to determine whether there are significant regional variations in urinary calculus composition and, if so, whether these correlate with any tap water variable since different types of calculi may be impacted in different ways by different types of water. In the future, it would also be interesting to perform additional epidemiological studies, in particular ecological studies related to water composition and incidence of KSD.

## 5. Conclusions

The mineral content of tap water varies significantly between different regions in the United Kingdom. Depending on where one lives, drinking 2–3 L of tap water per day can contribute over one-third of recommended daily calcium and magnesium intake, making tap water a significant but often overlooked source of these minerals. Whilst the exact relationship between drinking water mineral content and KSD incidence and recurrence has yet to be fully elucidated, clinicians should be mindful that in some regions, tap water can be a significant source of important minerals such as calcium, especially when counselling patients already on supplementation for other medical conditions. Future studies should focus on tailoring preventative strategies related to fluid consumption to the type of drinking water available to patients, 24-h urine chemistries and calculus composition to deliver more effective, personalised preventative strategies for patients at risk of recurrence.

## Figures and Tables

**Table 1:** Comparison of water composition by country in the United Kingdom.

Country	Median Total Hardness/mg/L [Range]	Median Calcium Concentration/mg/L [Range]	Median Magnesium Concentration/mg/L [Range]	Median Sodium Concentration/mg/L [Range]	Median Sulphate Concentration/mg/L [Range]
*England*	192.90 [19.00–331.50]	77.56 [6.73–128.00]	4.65 [1.30–31.80]	17.90 [2.98–57.80]	37.00 [7.95–112.43]
*Scotland*	32.87 [16.02–52.38]	10.69 [5.35–17.73]	1.59 [0.65–2.02]	6.39 [3.93–8.23]	9.07 [2.86–18.38]
*Wales*	68.93 [26.15–124.29]	26.83 [8.98–36.93]	3.88 [0.59–7.14]	10.93 [5.99–22.70]	6.74 [6.74–37.30]
*Northern Ireland*	100.95 [24.40–141.50]	31.50 [8.60–56.60]	5.35 [0.70–8.90]	N.D.	N.D.
Total	120.59 [16.02–331.50]	30.46 [5.35–128.00]	3.62 [0.59–31.80]	14.72 [2.98–57.80]	25.36 [2.86–112.43]

N.D. denotes no data available.

**Table 2:** Pairwise t-test significance values for differences in mean water variables by country in the United Kingdom.

Total Water Hardness				
	*England*	*Wales*	*Scotland*	*Northern Ireland*
*England*		**0.001**	**<0.001**	**0.009**
*Wales*	**0.001**		**0.005**	0.460
*Scotland*	**<0.001**	**0.005**		**0.003**
*Northern Ireland*	**0.009**	0.460	**0.003**	
**Calcium**				
	*England*	*Wales*	*Scotland*	*Northern Ireland*
*England*		**0.016**	**0.001**	**0.044**
*Wales*	**0.016**		**0.006**	0.381
*Scotland*	**0.001**	**0.006**		**0.005**
*Northern Ireland*	**0.044**	0.381	**0.005**	
**Magnesium**				
	*England*	*Wales*	*Scotland*	*Northern Ireland*
*England*		0.283	**0.048**	**0.044**
*Wales*	0.283		**0.020**	0.443
*Scotland*	**0.048**	**0.020**		**0.010**
*Northern Ireland*	**0.492**	0.443	**0.010**	
**Sodium**				
	*England*	*Wales*	*Scotland*	*Northern Ireland*
*England*		0.057	**0.001**	N.D.
*Wales*	0.057		**0.011**	N.D.
*Scotland*	**0.001**	**0.011**		N.D.
*Northern Ireland*	N.D.	N.D.	N.D.	
**Sulphate**				
	*England*	*Wales*	*Scotland*	*Northern Ireland*
*England*		**0.041**	**0.001**	N.D.
*Wales*	**0.041**		**0.033**	N.D.
*Scotland*	**0.001**	**0.033**		N.D.
*Northern Ireland*	N.D.	N.D.	N.D.	

N.D. denotes no data available; *p* values in bold are statistically significant at the 0.05 level.

**Table 3:** Comparison of water composition by region in England.

Region	Median Total Hardness/mg/L [Range]	Median Calcium Concentration/mg/L [Range]	Median Magnesium Concentration/mg/L [Range]	Median Sodium Concentration/mg/L [Range]	Median Sulfate Concentration/mg/L [Range]
*East*	269.00 [182.28–331.50)	104.90 [78.10–128.00]	8.74 [2.07–17.10]	28.40 [10.10–57.80]	59.79 [20.00–111.00]
*East Midlands*	165.90 [148.25–192.80]	59.30 [59.30–59.30]	31.80 [31.80–31.80]	29.80 [25.00–37.00]	64.00 [50.00–87.60]
*London*	272.00 [271.00–273.00]	N.D.	4.30 [4.20–4.30]	28.60 [27.60–29.60]	47.35 [46.40–48.30]
*North East*	116.88 [50.03–277.43]	38.05 [16.67–77.01]	5.29 [2.03–28.33]	17.33 [7.98–43.35]	61.35 [29.87–112.43]
*North West*	70.00 [19.00–109.00]	17.90 [6.73–33.60]	2.49 [1.30–6.13]	14.50 [2.98–19.00]	26.10 [7.95–42.80]
*South East*	285.75 [257.00–302.00]	104.72 [97.00–112.43]	4.80 [3.00–7.98]	14.93 [9.03–42.90]	20.29 [12.91–109.00]
*South West*	185.30 [39.98–33-.38]	97.085 [62.00–119.66]	6.30 [4.50–8.10]	10.60 [8.20–45.30]	14.60 [9.00–86.00]
*West Midlands*	156.25 [51.10–212.40]	27.58 [27.58–27.58]	3.10 [3.10–3.10]	16.60 [11.10–35.90]	34.65 [21.00–64.00]
**Total**	**192.80 [19.00–331.50]**	**77.56 [6.73–128.00]**	**4.65 [1.30–31.80]**	**17.90 [2.98–57.80]**	**37.00 [7.95–112.43]**

N.D. denotes no data available.

**Table 4:** Comparison of bottled and tap water in the United Kingdom.

	Median Calcium Concentration/mg/L [Range]	Median Magnesium Concentration/mg/L [Range]	Median Sodium Concentration/mg/L [Range]	Median Sulphate Concentration/mg/L [Range]
Bottled still [8]	55.00 [12.00–59.00]	10.05 [3.50–19.00]	11.90 [7.03–12.00]	12.00 [9.00–14.00]
Bottled sparkling [8]	56.00 [55.00–104.00]	18.00 [10.00–19.00]	11.50 [7.47–24.00]	13.00 [9.00–28.00]
Tap water	30.46 [5.35–128.00]	3.62 [0.59–31.80]	14.72 [2.98–57.8]	25.36 [2.86–112.43]

## Data Availability

Data generated and analysed are included in this study. Further enquiries can be directed to the corresponding author regarding acquisition of water quality reports used in this investigation.
